# Neural Evidence of Superior Memory: How to Capture Brain Activities of Encoding Processes Underlying Superior Memory

**DOI:** 10.3389/fnhum.2019.00310

**Published:** 2019-09-04

**Authors:** Jong-Sung Yoon, Jeremy Harper, Walter R. Boot, Yanfei Gong, Edward M. Bernat

**Affiliations:** ^1^Department of Psychology, University of South Dakota, Vermillion, SD, United States; ^2^Department of Psychology, University of Minnesota, Minneapolis, MN, United States; ^3^Department of Psychology, Florida State University, Tallahassee, FL, United States; ^4^Shanghai Academy of Educational Sciences, Shanghai, China; ^5^Department of Psychology, University of Maryland, College Park, College Park, MD, United States

**Keywords:** superior memory, event-related potentials, protocol analysis, memory training, digit-span

## Abstract

Relatively little attention has been paid to the neural basis of superior memory despite its potential in providing important insight into efforts to improve memory in the general population or to offset age-related cognitive decline. The current study reports a rare opportunity to reproduce and isolate specific neural activities directly associated with exceptional memory. To capture the brain processes responsible for superior memory, we returned to a laboratory task and analytic approach used to explore the nature of exceptional memory, namely, digit-span task combined with verbal protocol analysis. One participant with average memory received approximately 50 h of digit-span training and the participant’s digit-span increased from normative (8 digits) to exceptional (30 digits). Event-related potentials were recorded while the participant’s digit span increased from 19 to 30 digits. Protocol analysis allowed us to identify direct behavioral indices of idiosyncratic encoding processes underlying the superior memory performance. EEG indices directly corresponding to the behavioral indices of encoding processes were identified. The results suggest that the early attention-related encoding processes were reflected in theta and delta whereas the later attention-independent encoding processes were reflected in time-domain slow-wave. This fine-grained approach offers new insights into studying neural mechanism mediating superior memory and the cognitive effort necessary to develop it.

## Introduction

Studies with brain-damaged individuals have enhanced our understanding of the functional anatomy and processes of human memory (Medina and Fischer-Baum, 2017). However, relatively little attention has been paid to the basis of superior memory despite its potential to offer important insight into efforts to improve memory in the general population (Maguire et al., 2003) or to offset age-related cognitive decline. Perhaps most fundamentally, this is because reproducing and capturing mechanisms underlying superior memory under controlled laboratory or field situations is a difficult task for investigators. Furthermore, when researchers use brain-imaging techniques, it is particularly critical to identify and capture neural activity directly corresponding to a specific event while one engages in a task involving a continuous stream of complex cognitive processes (Spiers and Maguire, 2007a,b; Maguire, 2012).

The present article reports a rare opportunity that allows us to reproduce and directly investigate specific brain activities reflecting essential encoding processes of superior memory. In order to capture key neural processes hypothesized to underlie superior memory, the current study returned to a laboratory task and analytic approach used to discover the existence of and explore the nature of exceptional memory, namely, the digit span task combined with verbal protocol analysis (Ericsson and Simon, 1993). Protocol analysis of verbal reports of experts’ thoughts has widely been used to trace and identify essential mechanisms underlying their superior performance, including exceptional memory skill (for a review, Ericsson, 2006). We applied verbal protocol analysis to the well-understood process of developing complex mnemonics underlying the encoding and retrieval of serially presented digits during a sustained training process. Specifically, in the current study, a single participant with baseline average memory engaged in approximately 50 h of digit-span training. We observed the participant’s digit-span increase from normative (8 digits) to exceptional (30 digits) as a result of this training. More interestingly, while the digit-span increased from 19 digits to 30 digits, we collected event-related potentials (ERPs) during the majority of the training process. Then, by combining ERPs with direct behavioral evidence of encoding processes revealed by protocol analysis, the current study successfully captured important brain-based correlates associated with superior memory performance.

### Studies on the Development of Superior Memory and Protocol Analysis

Superior memory going beyond the working memory capacity can be explained by the acquisition of elaborate encoding and retrieval schemes in long-term memory, which requires extensive amounts of practice at the limits of one’s performance (Ericsson and Lehmann, 1996; Ericsson, 2003). However, when individuals display superior memory, it is very difficult to study if and how the memory performance changed during its development and whether only some select people can develop such a performance. In the late 1970s, to address the questions, Ericsson et al. (1980) examined the effects of digit-span training on two college students whose initial digit-spans were in the normal range (7 ± 2 digits). After several 100 h of practice, both participants became capable of recalling over 80 digits in the digit-span task. More importantly, the investigators collected participants’ verbal reports on their thought process after most memory trials and conducted a protocol analysis of the verbal reports (Ericsson and Simon, 1993). This allowed the researchers to discover the cognitive mechanisms mediating the increases in digit-span. Specifically, the development of participants’ idiosyncratic mnemonic skills was successfully identified based on information revealed by the protocol analysis. In the beginning of training, participants simply rehearsed the digits before an immediate serial recall of the digits. The major increase in digit-span was initiated by encoding groups of three or four digits with prior knowledge in long-term memory (LTM). For instance, the participants mainly encoded the digits as running times because they were avid cross-country runners (e.g., 358 was encoded as 3 min and 58 s). With extended practice, the participants were able to rapidly encode and store many digit groups in LTM and build unique retrieval structures by associating the encoded digits with mnemonic cues.

Based on these findings from a series of experiments of trained participants, Chase and Ericsson (1981, 1982) proposed *skilled memory theory* that successfully accounted for the acquisition of superior memory performance in various domains. The theory was later expanded into *long-term working memory* (LTWM) *theory* to explain how experts with no general memory superiority exhibited exceptional memory performance in their domain of expertise (Ericsson and Kintsch, 1995). Protocol analysis of participants’ verbal reports has been used as an essential technique to identify encoding and retrieval processes underlying participants’ mnemonic skills by capturing direct behavioral evidence of them, such as the effects of serial position (i.e., position of digit within each mnemonic group or position of each mnemonic group within a story) on study and retrieval times (Hu et al., 2009; Hu and Ericsson, 2012).

For instance, protocol analysis allowed Hu et al. (2009) to uncover the source of Chao Lu’s (C.L.), Guinness World Record holder for memorizing π at that time, exceptional memory skill. C.L’s memory was tested with a self-paced memorization task for random digits and protocol analysis showed that C.L. converted every two digits into a meaningful thing or person, mainly based on phonetic similarity. For instance, 79 was converted into a balloon because 79 is ‘qi jiu’ and a ballon is ‘qi qiu’ in Chinese. He would then use to generate vivid stories that would involve several 2-digit groups. For example, a digit sequence 8 7 1 1 7 9 was encoded as “A royal man (87) used the chopsticks (11) to stab a balloon (79).” As behavioral evidence in support of this mnemonic strategy, Hu et al. (2009) found that during the self-paced task C.L.’s study times of the digits at even serial positions were longer than those at the odd serial positions. This difference in study times supported a hypothesis that for each 2 digit group C.L. would encode the pair mnemonically into LTM. The reversed pattern was found for recall times because after the first digit (i.e., odd serial position) was retrieved the second digit (i.e., even serial position) could quickly be recalled based on association. Hu and Ericsson (2012) further found that C.L.’s study times even varied as a function of the serial position within the story. Specifically, C.L.’s study times on each 2-digit group increased as the number of digit groups within the story increased. He spent the longest time on the last digit group (i.e., a balloon in the story above) because he would need to spend more time for reviewing the story after the last digit group. During his recall, the pattern was also reversed because the first group could only be recalled after the retrieval of the story from LTM.

The present study was designed to replicate behavioral measures of superior memory performance at this same level of detail, and then assess brain-based measures of cognitive processes active during encoding.

### Brain-Imaging Studies of Superior Memory

In early 2000s, several brain-imaging studies demonstrated that individuals who showed exceptional performance going beyond a traditional WM capacity exhibited activity changes in certain brain regions compared to control groups (Pesenti et al., 2001; Tanaka et al., 2002; Maguire et al., 2003). Specifically, Maguire et al. (2003) recruited superior memoirists from the World Memory Championship and compared their patterns of brain activity [assessed via functional magnetic resonance imaging (fMRI)] and brain structures with those of control subjects. During memorization, the superior memory performers showed relative increases in brain regions associated with spatial memory (e.g., right posterior hippocampus) compared to the control group. This suggests that relative differences in regional brain activity were attributable to the unique encoding strategies used (e.g., method of loci) by superior memorists. Using electroencephalography (EEG) to record ERPs during a memory recognition task, Williamon and Egner (2004) found changes in a right-lateralized centroparietal ERP component associated with the encoding and retrieval of information in expert pianists. In Raz et al. (2009), a subject exhibiting an exceptional ability to memorize the mathematical constant π showed increased activity in several brain regions while recalling π with the method of loci, but activated different brain regions when asked to encode unfamiliar random digits. Most recently, Yin et al. (2015) examined differences in a pattern of brain activity between the former holder of *Guinness World Record* for reciting π (C.L.) and control subjects. Previous studies on C.L have revealed that C.L. grouped the digits of π into 2 digit-groups and associated them with images creating vivid stories of them (Hu et al., 2009; Hu and Ericsson, 2012). Given his mnemonic strategies, Yin et al. (2015) asked him and control subjects to study and recall 2-digit numbers, and hypothesized a stronger activity in the brain regions associated with C.L.’s mnemonic strategies. Consistent with the expectation, compared to the control subjects, C.L. showed a stronger activation in brain regions (e.g., frontal poles, left superior parietal lobule) associated with episodic memory while he used his mnemonic strategies for studying and recalling the 2-digit numbers. Most recently, Pan et al. (2017) collected EEG activities while superior mnemonists and control participants performed a digit memory task (12 digits). They found that the mnemonists generated mental images by 2-digit groups to process the 12-digit number. Consistent with the mnemonic strategy, only the mnemonists showed an increased P2 component and high-alpha oscillation on digits at even positions compared to digits at odd-position.

These results have supported the notion that exceptional memory performance is a result of the development of elaborate encoding and retrieving processes. However, it has been difficult to isolate a neural activity corresponding to a specific encoding or retrieval process underlying superior memory from many other cognitive processes while the subjects were asked to perform memory tasks in a few seconds. Spiers and Maguire (2007b) attempted to address a similar methodological concern by combining a technique of protocol analysis (Ericsson and Simon, 1993) with fMRI (for a further review, see Spiers and Maguire, 2007a; Maguire, 2012). The results provide evidence that collecting a retrospective verbal report of participants’ thoughts can allow researchers to pinpoint a meaningful cognitive process underlying behavior and to associate it with a specific brain activity at that time.

### The Present Research

**FIGURE 1:**
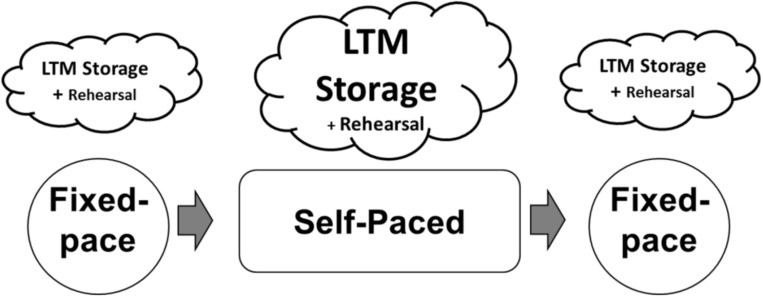
Design of study and expected differences in memory processes by block. It was expected that the participants would mostly encode digits into meaningful group to be stored in LTM during fixed-pace and self-paced tests, with a rote rehearsal strategy for digits presented late in each trial sequence. The encoding processes (for LTM storage) were anticipated to be larger (so it would be easier to capture) in the self-pace test compared to the fixed-pace test because of the additional time available for meaningful digit groups.

In this study, one participant with baseline average memory abilities participated in approximately 50 h of training designed to increase digit-span by applying the mnemonic system developed by the trained participants in Chase and Ericsson (1981, 1982). The basic design of each training session and differences in essential memory processes by blocks are summarized in Figure 1. Each session consisted of two blocks of fixed-presentation rate digit-span task (Fixed-paced test) and a block of self-paced digit-span task (Self-paced test). Employing a self-paced test, where the participant is allowed to regulate the presentation rate of digit sequences, was one of the essential methodological techniques in this study. This is because it would allow us to replicate and capture a direct behavioral evidence of encoding process underlying participant’s idiosyncratic mnemonic skills (i.e., the variation of study times) based on information revealed by a protocol analysis of verbal reports (e.g., Hu et al., 2009; Hu and Ericsson, 2012). Then, we combined the behavioral evidence of encoding process with ERPs in order to assess neural processes associated with superior memory performance (i.e., exceptional digit-span). It was expected that the participant would mostly encode digits into meaningful groups to be stored in LTM during fixed-pace and self-paced tests, with a rote rehearsal strategy for digits presented late in each trial sequence. The encoding processes (for LTM storage) were anticipated to be larger (so it would be easier to capture) in the self-pace test compared to the fixed-pace test because of the additional time available for meaningful digit groups.

It should be also noted that both fixed-paced and self-paced tests were designed to present a constant state of challenge so that the participant would attempt to perform at the limits of his/her performance all the time. In Chase and Ericsson (1981, 1982) original studies, the length of random digit sequence during the digit-span task was determined by the following rule: if participants successfully recalled a digit list on a given trial, they received a digit list that was one digit longer in the next trial; but if they failed, they received a digit list that was one digit shorter. By adopting the ±1 rule to determine the length of next trial, the digit-span task was constantly adapted to make the participant keep challenging their current level of performance. In our study we also made our digit-span tasks to present a constant state of challenge by applying the ±1 rule (in the fixed-paced task) or giving three digit longer than the current digit-span (in the self-paced task) based on the assumption that it would keep the participant at the limit of current digit-span during the entire training session.

Following this approach, we expected that the participant’s digit-span would significantly increase with training by associating digits with prior knowledge in LTM, as demonstrated in the previous studies (e.g., Chase and Ericsson, 1981, 1982; Hu et al., 2009). Particularly, given the anticipation that the participant would generate digit-groups with semantic codes and create a meaningful story combining them, as C.L. did in Hu et al. (2009), we specifically focused on collecting two essential pieces of information: (1) position of individual digit within each mnemonic group to identify first and last digits of each mnemonic group and (2) position of digit group within each story to identify first and final digit-groups of the story. The second piece of information allows the identification of whether the first digit or the last digit of each group was also the first digit or the last digit at the story level (i.e., super-group first digit or super-group last digit; Hu and Ericsson, 2012).

Based on these two essential pieces of information, we could set up two testable predictions regarding how the participant would segment and encode digits during the self-paced test. First, we predicted the participant would spend more times on the last digit of each group because it would be the moment when the participant would encode digit groups mnemonically into LTM as C.L. did for the second digit in his two digit groups. Second, the study time of the last digit of a group would be particularly prolonged if this digit were the last digit of the story (i.e., when the last digit was the super-group last digit), because the participant would consolidate the story by reviewing it after the last digit group (Hu and Ericsson, 2012). Third, we expected the patterns of study times would be reversed for the retrieval times as demonstrated in Hu et al. (2009) and Hu and Ericsson (2012). It is also important to notice that such difference in study time would not be observable during the fixed-paced test because the participants would not be allowed to regulate a presentation-rate.

Then we assessed for brain processes directly associated with the behavioral evidence of participant’s unique encoding process by linking it with ERPs corresponding to the digit encoding. First, because we anticipated study time difference between first and last digits, we expected to observe ERP differences between the first and last digits of digit groups during the self-paced test. Second, although it would be not possible to observe the study time differences in fixed-presentation rate, we anticipated a similar digit-position effect on EEG measures during the fixed-pace test given that the participant would essentially apply the same mnemonic technique across digit-span tasks. Third, we also examined a hypothesis that brain activities associated with the participant’s encoding process would be greater during the self-paced test compared to the fixed-pace test. In other words, given that the self-paced test would permit the participant to solely focus on each step of encoding processes without time pressure, encoding process for generating meaningful digit-groups might be more acutely active – relative to other ongoing cognitive processes – during the self-paced test.

It should be noted that the current study assumed the qualitative differences between the first and the last digits as hypothesized above. Thus the following statistical analyses were also conducted based on the assumption of independence between them although the observation was based on a single-participant.

## Materials and Methods

### Participant, Materials, and Procedures

The participant was a visiting graduate student from China at a Florida State University. She was 25-years old when testing began and typically tested 1–3 times a week over the 10 month period. She received 50 sessions of testing and each session took approximately 1 h. Written informed consent was obtained from the participant both for the purposes of research participation as well as for the publication of this study data. The study protocol was reviewed and approved by FSU Human Subjects Committee Institutional Review Board. All study procedures also adhered to standard biosecurity and institutional safety procedures. E-prime software (Psychology Software Tools, Inc., Pittsburgh, PA, United States) was used to administer the experimental procedure and present the digit-span tasks.

At the beginning of the first day, the participant was instructed to memorize random digit sequence as best as she could and was told that she would receive two different types of digit memorization tasks: (a) a fixed-paced test and (b) a self-paced test. During the fixed test-paced test, for each trial, random digits were presented at a rate of one digit per second (500 ms blank screen followed by 500 ms digit presentation). During the fixed-pace test, following the last digit of each sequence, the participant was asked to recall the presented digit sequence by typing it on a standard computer keyboard. Immediately after completing recall, the participant was asked to give a verbal report of thoughts during the trial. After the collection of verbal report, the participant was given feedback on the accuracy of recall (either ‘Correct’ or ‘Incorrect’ displayed centrally on the screen for 1000 ms). Except for the very first day of training (which started with a length of 4 digits), the length of digit sequence for the first trial was determined based on the length of the last trial of the fixed-pace test from the previous session.

After the first fixed-paced test, the participant was instructed to memorize digit sequence in the self-paced test. The self-paced test was nearly identical to the fixed-pace test except for two key differences. First, the participant was allowed to regulate the presentation rate of the digit sequence (i.e., time to study each digit before the next presented digit) by pressing the spacebar on the keyboard, which resulted in a variable study (encoding) time for each digit depending on the participant’s mnemonic strategy. Each digit presentation began with a blank screen (1000 ms), followed by a random digit; however, unlike the fixed-pace test, the participant had to press the spacebar to receive the next digit. There was no time limit for the study of a given digit. The study time was recorded for each of the digits by measuring the time from the digit onset to the next key press (which would initiate the next digit presentation). Second, the length of the digit sequences presented during each block of the self-paced test was three digits longer than the participant’s estimated digit-span at that time. The differences between the self-paced test and the fixed-paced test were instructed to the participant before starting the self-paced test on the first day. As illustrated in Figure 1, for each session, the participant was asked to perform another fixed-paced test after the self-paced test.

At the end of each test block, the participant was also asked to recall as much of the digit sequences presented as the participant could. But, the post-block recall performance was not analyzed in the current study because it fell beyond the scope of the current study. EEG data were recorded only for the last 16 sessions, which coincided with the participant’s digit span increasing from 19 to 30 digits. Thus, except for the digit-span data across all 50 sessions, only the data from those 16 sessions were used for the following EEG analyses. For each test block, the participant received trials of random digit sequences for either 10 (during the self-paced test) or 15 min (during the fixed-paced test). The E-prime program was designed to present trials of random digit sequences as many as possible during those minutes. Thus, as the participant’s digit-span increased, the number of trials presented in each test block tended to decrease (because the number of digits that needed to be presented and recalled in each trial increased). Specifically, the number of trials for the fixed-pace test during the last 16 sessions ranged from 11 to 4. The number of trials for the self-paced test ranged from 5 to 2. During the last 16 sessions, the participant additionally received a block of a control task before and after each of the fixed-pace test. The control task was identical to the fixed-pace test except that (1) the length of digit sequences were fixed at 6 digits and (2) each block of the control task consisted of 10 trials. The participant was told that there would be only 6 digits in each trial, and that the idea was to use simple rote memorization, rather than more complex techniques to recall them. It should be noted that we decided not to include the data from the control task in the current study since these control blocks involved a qualitatively different strategy (i.e., simple rehearsal of the 6 digits in WM). Instead we focus on neural and behavioral signatures of two types of blocks (fixed vs. self-paced), which would be directly associated with encoding processes of the participant’s unique mnemonic strategy.

For the following analyses, only the digits from correct trials were included. However, it should be noted that there were some correct trials in which the participant backspaced to correct earlier digits that were initially either skipped to recall or incorrectly recalled. Those correct trials were excluded for the analysis of retrieval times because the retrieval times of those digits (correctly recalled later) would be particularly prolonged regardless of digit position. The very first or the very last digits in a given trial were also excluded because either the retrieval or recall time for them would include times for reviewing every digit presented in a given trial.

### EEG Acquisition and Processing

Recordings were collected with a 128-channel Synamps RT amplifier (Neuroscan, Inc.) in conjunction with Neuroscan 128-channel Quik-Caps (sintered Ag-Ag/Cl). Ten electrodes located around the ears (five on each side) were removed offline due to consistently inadequate scalp connection, resulting in 113 electrodes available for analysis. Horizontal and vertical electrooculogram activity was recorded from electrodes located on the outer canthus of both eyes, and above and below the left eye, respectively. Signals were recorded using an analog 0.05 to 200 Hz bandpass filter at a sampling rate of 1000 Hz. Impedances were kept below 10 kΩ. All EEG signals were offline referenced to averaged mastoid signals.

Three-second epochs were taken from the continuous data from 1000 ms pre- to 2000 ms post-stimulus and baseline corrected with the 150 ms prestimulus activity. Epochs were corrected for ocular artifacts using an algorithm implemented in Neuroscan Edit 4.5 (Semlitsch et al., 1986; Neuroscan, Inc.). Signals were downsampled to 128 Hz using the Matlab resample function (Mathworks, Inc.), which applies an anti-aliasing filter during resampling. A two-step automated process for trial-level cleaning was performed: (1) whole trials were rejected if activity at F3 or F4 exceeded ±100 μV in either the pre- (−1000 to −1 ms) or post-stimulus (1 to 2000 ms) time windows (relative to one another), and (2) within-trial individual electrodes were rejected if activity exceeded ±100 μV based on the same pre- and post-stimulus time regions. Using this method, 13% of all data was removed. Signals were then lowpass filtered at 30 Hz using a 3^rd^ order Butter-worth filter and averaged according to block (self-paced, fixed-pace) and digit-position (1^st^ digit, Last digit). Following this procedure, visual inspection of the condition averaged signals indicated that 37 electrodes (out of 1808) became disconnected during recording and were removed from analysis.

### EEG Analysis

#### Time-Domain Components

**FIGURE 2:**
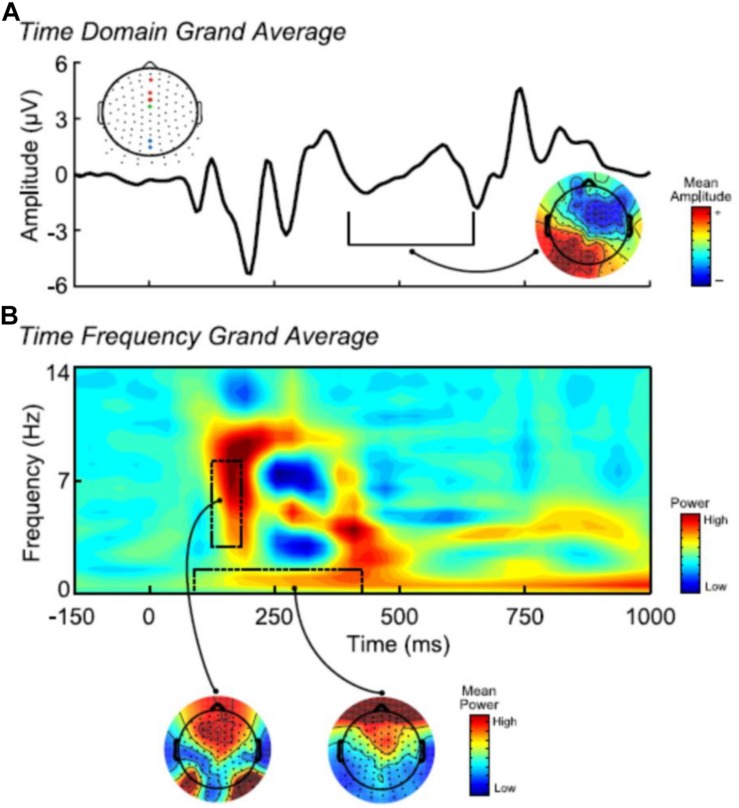
**(A)** Averaged stimulus-locked (time = 0) ERP across the first and last lower-level group digits. Dashed lines denote the time window for the slow-wave component (398–648 ms). The spatial map depicts the grand averaged topographic distribution of the slow-wave. The schematic topographic layout of the NeuroScan 128-channel non-standard layout Quik-Cap highlights the clusters of channels used for plotting and statistical analyses (Blue: slow-wave; Red: delta; Green and Red: theta). **(B)** Averaged time-frequency representation of the ERP across the first and last digits. The black dashed boxes denote the regions of interest for theta (3–8 Hz; 125–156 ms) and low-frequency delta (≤ 1 Hz; 94–406 ms). The spatial maps depict the grand averaged topographic distribution of theta and delta power.

Event-related potentials component measures were identified via visual inspection of the grand average waveforms. Six components were identified based on the peaks and troughs of the ERP, and were labeled based on peak polarity (positive, negative) and latency (Figure 2): (1) P120: peak positive deflection between 94 and 210 ms averaged across three midfrontal sites; (2) N210: peak negative deflection between 125 and 250 ms averaged across four midfrontal sites; (3) P250: peak positive deflection occurring between 211 and 289 ms averaged across six right parietal-occipital sites; (4) N290: peak negative deflection between 250 and 344 ms averaged across four midfrontal sites; (5) P350: peak positive deflection occurring between 289 and 398 ms averaged across three midline parietal sites; and (6) late slow-wave: mean positive potential between 398 and 648 ms at two midline parietal sites. Electrodes for statistical analysis were chosen based on the grand average topography (i.e., averaged over condition and digit position) for each ERP and time-frequency component. We particularly used the electrodes from the regions where activity was maximal for each component.

#### Time-Frequency Components

Time-frequency analysis was performed according to previous reports (Bernat et al., 2005, 2007, 2015; Hall et al., 2007; Harper et al., 2014, 2016). Trial-averaged ERPs were transformed into time-frequency energy representations using the binomial reduced interference distribution (binomial-RID), with −1 to 2 s epochs, resampled to 32 Hz (we assessed data up to 12 Hz), and with 2 frequency bins per Hz (0.5 Hz steps). Analyzing the TF dynamics of the condition-averaged signals allows for the representation of the phase-consistent EEG activity contained in the time-domain ERP in time-frequency space, which can be used to evaluate the TF dynamics directly related to the time-domain ERP components. The binomial RID benefits from uniform high-resolution time and frequency representation, improving estimation of time- frequency activity (e.g., relative to other TF decomposition methods such as wavelets; Bernat et al., 2005; Aviyente et al., 2011). Four regions-of-interest (ROI) were identified via visual inspection of the grand average TF representation (all calculated as the average activity within the ROI): (1) theta activity between 125–156 ms and 3–8 Hz at four midfrontal sites; (2) low-frequency delta activity between 94–406 ms and ≤1 Hz at three midfrontal sites; (3) alpha activity between 94–281 ms and 9–10.5 Hz at three midfrontal sites; and (4) delta activity between 344–406 ms and 1.5–3 Hz at four midfrontal sites. We did not focus on TF measurement of late slow-wave activity (i.e., >500 ms), as this activity contains primarily low-frequency activity, uncomplicated by the ERP components in the first 500 ms, and is thus well-represented in the time-domain measures.

Previous research has indicated that many time-domain ERP components can be understood as temporally superimposed activity from several frequency bands (e.g., Başar, 1980; Başar-Eroglu et al., 1992; Karakaş et al., 2000; Harper et al., 2014; Bernat et al., 2015). Following these approaches, regression analyses were conducted to assess whether the present TF components would more parsimoniously account for the early ERP component measures (i.e., excluding the late time-domain slow-wave potential, for which we used the time-domain measure as described above). Separate models were fit for each TD component as the dependent variable, and the TF measures that co-varied in time with the TD component served as predictors. Results indicated that for each ERP measure, the associated TF measures together significantly explained a majority of the variance (*R*^2^ range: 0.53–0.62). For P120 (*R*^2^ = 0.62), theta and low-frequency delta were uniquely predictive (*p* < 0.05), but alpha was not (*p* = 0.28). For N120 (*R*^2^ = 0.56), low-frequency delta was uniquely predictive (*p* < 0.01), but theta and alpha were not (*p* = 0.57, 0.18, respectively). For P250 (*R*^2^ = 0.56), low-frequency delta was uniquely predictive (*p* < 0.01), but alpha was not (*p* = 0.43). For N290 (*R*^2^ = 0.56), low-frequency delta (*p* < 0.05), but not delta (*p* = 0.97), was significantly predictive. When predicting P350 (*R*^2^ = 0.53), delta and low-frequency delta were not significantly predictive (*p* = 0.26, 0.28, respectively), but the two TF measures were highly collinear (*r* = 0.82) and there were significant zero order correlations between the two TF measures and the P350 (*r* = −0.69, −0.68, respectively, *ps* < 0.01). Taken together, these models indicate that the activity underlying the early time-domain ERP components can be best indexed using TF theta and low-frequency delta occurring throughout the early ERP response. Thus, we used TF theta and low-frequency delta (hereinafter referred to as delta for convenience) measures to index the early ERP activity, and the time-domain slow-wave (TD-SW) measure to index the late activity. Figure 2 depicts the ERP and TF measures of interest. These three measures served as the units of analysis in the statistical models described below.

## Results

### Exceptional Memory Performance and Protocol Analysis

**FIGURE 3:**
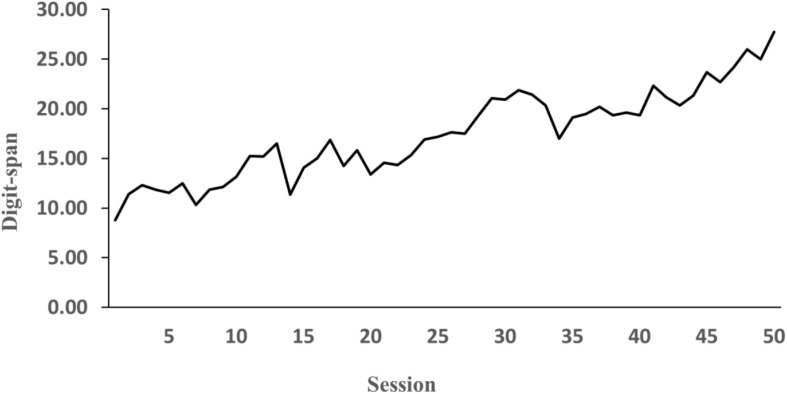
Participant’s average digit-span during 50 sessions of practice.

Consistent with previous studies, performance on the fixed-pace test was used to calculate the participant’s digit-span. Over the course of 50 h, the participant’s digit-span increased from unexceptional levels (9 digits) to exceptional levels of performance (30 digits) as shown in Figure 3. As expected, the protocol analysis of verbal reports showed that the participant developed an idiosyncratic mnemonic system. During both the fixed-pace test and the self-paced test the participant grouped the digits with various semantic codes that already existed in the participant’s LTM. For instance, a digit sequence 1 7 6 0 8 9 3 was encoded as “*the 1 m 76 cm tall man (176) joined Jiu San Society (93) [A Chinese political party; 93 is also ‘jiu san’ in Chinese] in the year 08 (08).*” In this example, there were three digit groups (i.e., the 1 m 76 cm tall man, Jiu San Society, and the year 08) in this sequence and the three groups combined as one story or super-group (i.e., the 1 m 76 cm tall man joined Jiu San Society in the year 08). The protocol analysis allowed us to differentiate the first digits of digit groups from the last digits and identify if those first and last digits were the super-group first and last digits. For example, in the same sequence, 1, 0, and 9 were the first digits of each group whereas 6, 8, and 3 were the last digits. Particularly, 3 was the super-group last digit and 1 was the super-group first digit in the sequence.

### Behavioral Indices of Encoding Process: Effects of Digit-Position on Study and Retrieval Times in the Self-Paced Test

**FIGURE 4:**
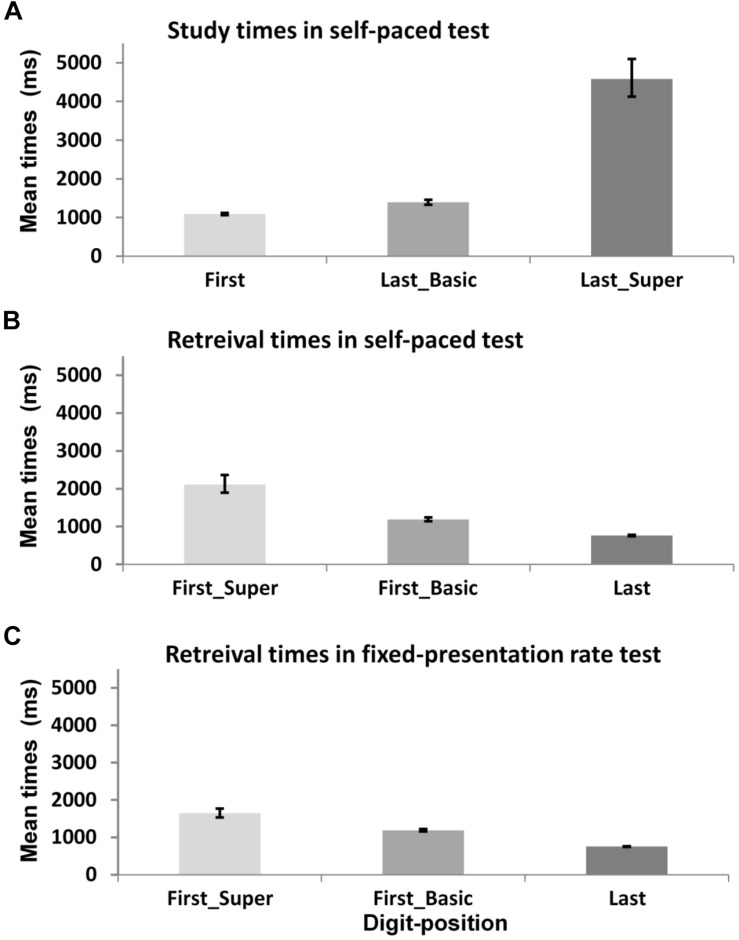
Mean study and retrieval times for different digit-position within each mnemonic group. Each panel shows digit-position effect on **(A)** study times in self-paced test, **(B)** retrieval times in self-paced test, and **(C)** retrieval times in fixed-presentation rate test. Error bars represent ±1 SE.

Before conducting the following analyses, a log transformation was applied to address a positive skewness in the distribution of study and retrieval times. For ease of interpretation, raw (anti-log transformed) means are presented in brackets using the unit of milliseconds (untransformed values are also used in Figure 4). We successfully found direct behavioral evidence of the participant’s unique encoding process by linking the digit-position information – which was revealed in the protocol analysis above – with the study times for digits in the self-paced test. First, as illustrated in Figure 4A, the study times of each digit varied as position of individual digit within each mnemonic group. The study times for the last digit of basic-groups (*M* = 2.90 [1,394 ms], *SD* = 0.58) were significantly longer than those for the first digit (*M* = 2.69 [1089 ms], *SD* = 0.48), *t*(607) = 4.97, *p* < 0.001, *d* = 0.4. Study times were especially prolonged for last digits of super-groups (*M* = 3.60 [4,581 ms], *SD* = 0.55) compared to the last digits of basic-groups (*M* = 2.90 [1,394 ms], *SD* = 0.58), *t*(371) = 10.71, *p* < 0.001, *d* = 1.2.

Second, there was also an effect of digit-position on the retrieval times of each digit in the self-paced test; specifically the pattern of retrieval times was a reversal of the pattern of study times as shown in Figure 4B. Retrieval times for the first digit of basic-groups (*M* = 2.77 [1,189 ms], *SD* = 0.43) were significantly longer than those for the last digit (*M* = 2.21 [762 ms], *SD* = 0.60), *t*(305) = 9.59, *p* < 0.001, *d* = 1.07. And the retrieval times of the first digit of super-groups (*M* = 3.18 [2,114 ms], *SD* = 0.43) were significantly longer than those of basic-groups (*M* = 2.77 [1,189 ms], *SD* = 0.43), *t*(172) = 5.27, *p* < 0.001, *d* = 1.39. Further, the same pattern of retrieval times was found in the fixed-paced test as shown in Figure 4C. Retrieval times of the first digit of basic-groups were significantly longer (*M* = 2.77 [1,189 ms], *SD* = 0.45) than those of the last digit (*M* = 2.19 [755 ms], *SD* = 0.59), *t*(704) = 14.9, *p* < 0.001, *d* = 1.45, and retrieval times of the first digit of super-groups (*M* = 3.02 [1,647 ms], *SD* = 0.40, *SE* = 0.049) were even longer than those of basic-groups (*M* = 2.77 [1,189 ms], *SD* = 0.45), *t*(368) = 4.34, *p* < 0.001, *d* = 0.59.

### Corresponding Electrocortical Indices: Effects of Digit-Position and Interactions With Presentation-Pace and Measures

**FIGURE 5:**
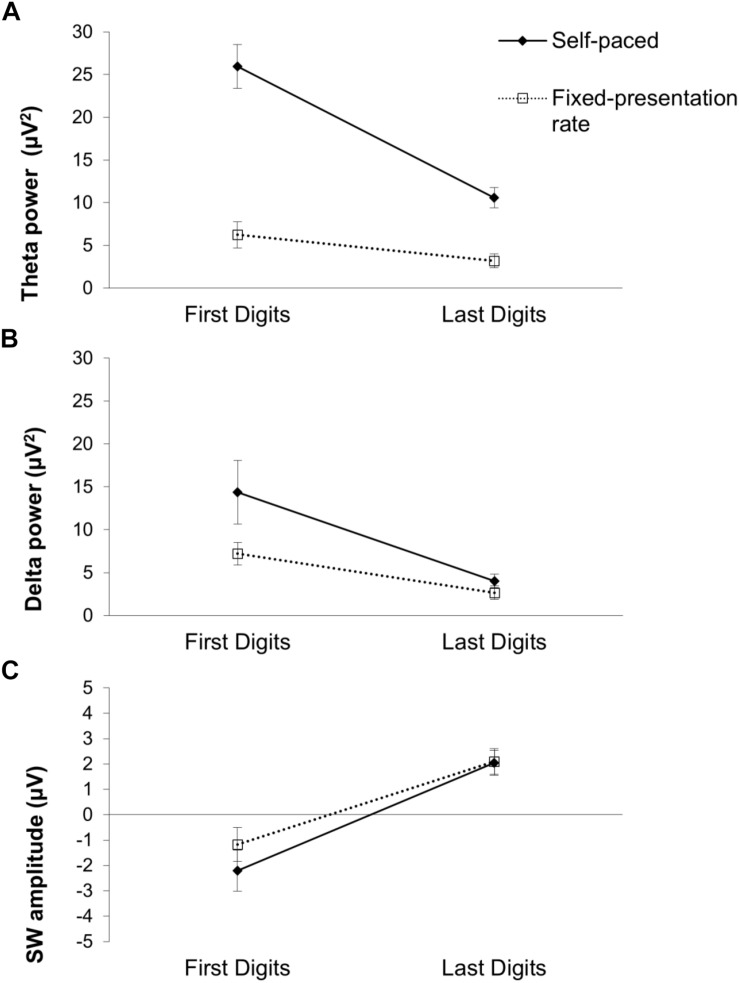
Means of **(A)** theta power, **(B)** delta power, **(C)** SW amplitude by the digit-position and the presentation-rate. Error bars represent ±1 SE.

To find ERP differences corresponding to the behavioral indices above, we first examined the effects of digit-position on EEG measures separately for the self-paced and the fixed-paced test conditions. In the self-paced test, as shown in Figure 5, the early EEG components (theta, delta) were greater for the first digits than for the last digits of digit groups [theta, *t*(15) = 6.25, *p* < 0.001, *d* = 1.56; delta, *t*(15) = 2.84, *p* < 0.05, *d* = 0.71]. A significant difference between the first and the last digits in the later TD-SW amplitude (i.e., negative TD-SW for the first digits and positive TD-SW for the last digits) was also observed, *t*(15) = 6.65, *p* < 0.001, *d* = 1.66. A similar pattern of significant effects of digit-position was observed in the fixed-pace test [theta, *t*(15) = 2.19, *p* < 0.05, *d* = 0.55; delta, *t*(15) = 3.38, *p* < 0.01, *d* = 0.85; TD-SW, *t*(15) = 4.82, *p* < 0.01, *d* = 1.21]. The results indicated that qualitatively similar processing mechanisms occurred during the self-paced test and the fixed-pace test, consistent with our hypothesis that the participant would use the same mnemonic technique across digit-span tasks as indicated also by the verbal protocol analysis.

In order to test whether the ERP and time-frequency component digit position differences were modulated by presentation-pace, we conducted a 2 (digit position: first, last) × 2 (presentation-pace: self-paced, fixed-pace) repeated-measures ANOVA separately for each EEG measure. The results of the two-way ANOVAs are presented in Table 1. First, the robust digit-position effects were replicated across all three EEG measures (as described above). Second, a significant main effect of presentation-pace was only found for time-frequency theta and delta, indicating that theta and delta power increased during the self-paced test compared to the fixed-pace test, and this pattern was particularly prominent for theta. In contrast, the main effect of presentation-pace was not significant for the TD-SW. Third, an interaction between digit-position and presentation-pace was only found in theta, indicating that the first–last digit position effect was greater during the self-paced test than the fixed-pace test. This interaction was not significant for delta or TD-SW. These different patterns of presentation-pace effect and the interaction of digit-position and presentation-pace are illustrated in Figure 5. Taken together, these results suggest that cognitive processes reflected in TD-SW may be independent of the pacing and different from those reflected in the early EEG measures (theta and delta).

**TABLE 1:** Summary of two-way GLMs for each EEG measure.

	**Effects of digit-position**			**Effects of presentation-pace**			**Interaction**
	**First digit**	**Last digit**		**Self-paced test**	**Fixed test**		**Digit-position by Presentation-pace**
**Measure**	**(*M, SE*)**	**(*M, SE*)**	***F***	**(*M, SE*)**	**(*M, SE*)**	***F***	***F***
Theta	16.08 (1.68)	6.89 (0.80)	37.24^∗∗^	18.26 (1.58)	4.7 (1.01)	79.37^∗∗^	21.96^∗∗^
Delta	10.78 (2.04)	3.35 (0.65)	15.01^∗∗^	9.2 (1.96)	4.94 (0.84)	5.07^∗^	2.21
TD-SW	−1.69 (0.62)	2.08 (0.34)	60.87^∗∗^	−0.08 (0.59)	0.47 (0.49)	0.74	1.20

*^∗∗^*p* < 0.01, ^∗^*p* < 0.05.*

## Discussion

In the current study, we investigated brain processes directly associated with the behavioral evidence of an individual’s unique encoding process underlying superior memory. This novel study demonstrated how ERP measures related to memory encoding can be combined with ideographic behavioral indices of memory processes derived from the participant’s verbal reports of their thought process during encoding.

### Behavioral Indices of the Unique Encoding Skill

The participant successfully acquired superior memory with 50 h of digit-span training. The participant’s digit-span increased dramatically from 9 digits to 30 digits, which not only is far beyond the normal range of digit-span, but also surpasses some famous mnemonists, such as Luria’s (1968) S and Hunt and Love’s (1972) VP (Yoon et al., 2018).

The digit-position effect on study and retrieval times was found as an index of the participant’s idiosyncratic encoding skill underlying the superior memory performance. The protocol analysis of verbal reports allowed us not only to show that the participant grouped the presented digits with previously acquired knowledge in LTM, but also to collect the digit-position information. As soon as the first digit was presented, the participant would process it rapidly, generating possible mnemonic associations. On the other hand, when the last digit was presented, the participant needed more time to process it to form a digit-group and complete mnemonic association for it in LTM (Hu et al., 2009), maintaining and updating the digit(s) previously presented. Particularly, if it was the last digit of the final group in a corresponding story (super-group), the participant would spend additional time to finalize, review and consolidate the story for firmly encoding it into LTM (Hu and Ericsson, 2012). The pattern of digit position effect on study times corresponds to the differences in encoding process between the first and last digits. The reversed pattern of retrieval times (i.e., the longer retrieval times for the first digits) also supports the participant’s idiosyncratic encoding and retrieval techniques, which can be explained in the context of LTWM model as demonstrated in Hu et al. (2009) and Hu and Ericsson (2012). In sum, the behavioral indices of the participant’s mnemonic skill suggest that encoding the last digits would consist of a more complex stream of ongoing cognitive processes – compared to encoding the first digits. The results also indicate that the participant’s encoding would start off with a relatively clean-cognitive slate when it was the first digit – after a completion of encoding of a digit group at the end of a previous digit (i.e., the last digit).

### ERP Indices Corresponding to Encoding Process

The most striking finding in the current study is that digit-position (i.e., first or last digit of digit group mnemonically encoded) had a significant effect on the all EEG measures of interest. The finding indicates that key elements of highly ideographic meta-cognitive memory processes employed in the development of expert memory performance can be indexed using EEG neuroimaging approaches. Particularly, we believe that the EEG measures captured two different processes: (1) the early attention-related encoding processes reflected in theta and delta and (2) the later attention-independent encoding processes reflected in TD-SW.

#### Early Encoding Processes

Above all, theta and delta activity during early encoding were significantly greater for the first digits (compared to the last digit). This is consistent with the participant’s encoding process that would be initiated by increased attention (increases in amplitude) to the first digits, while the last digit garners reduced attention – presumably due to the additional processes associated with maintaining/updating previous digits in memory and mnemonic associations. Theta activity has been consistently linked to increased attention/orienting toward task-relevant stimuli and executive functioning processes (Cavanagh et al., 2012; for reviews, see Cavanagh and Frank, 2014; Clayton et al., 2015). It has also been suggested that increased theta power during memory processes is reflective of a need for increased top–down processes – such as expectancy and selective attention – for activating and selecting memory traces in LTM (for a review, Klimesch et al., 2008; Nyhus and Curran, 2010). Supporting this claim, increased theta power has been associated with successful information encoding and subsequent retrieval (Klimesch et al., 1997) and this finding has been widely replicated (for a review, see Nyhus and Curran, 2010).

Thus, the significantly greater activation of the early EEG measures during the first digits seems most likely to reflect the aspect of clean-cognitive slate for initiating encoding process of new digit group. That is, at the onset of the first digits, the participant would be more ready to encode a new digit-group, and would need to devote less cognitive effort toward other processes required for processing the last digits of a group (e.g., maintaining and updating previous digits, finalizing and reviewing mnemonic association). In other words, the theta activity related to top–down control might be more acute in the first digits than in the last digits because it was more isolated from the other processes. Previous work from our group has supported the view that theta tends to index a more simple process sensitive to the most primary aspects of the task stimuli, whereas lower frequency activity (delta) indexes both the primary aspects and an array of task specific secondary stimulus aspects requiring greater elaborative processing (Bernat et al., 2015; Harper et al., 2016). Given this early theta and delta activity is consistent with increased attentional processing for accessing and retrieving knowledge from LTM.

#### Later Encoding Processes

The late TD-SW component was also sensitive to the digit-position effect, showing a bi-directional pattern of activation depending on the digit-position, namely, a more negative slow-wave for the first digits and a more positive slow-wave for the last digits. Interestingly, the effect can be interpreted in different ways because it occurred in the later part of the participant’s encoding process. First, the negative TD-SW during the first digits could reflect preparatory process for the next digit, as has been suggested by others (e.g., McCallum, 1988; Rockstroh et al., 1993), and/or retention operations in WM (e.g., Ruchkin et al., 1995) for maintaining the presented digit(s). However, it is also interesting to note that the last digits were consistent with a different pattern of TD-SW (i.e., positivity). Given the TD-SW positivity during the last digits, an alternate explanation of the TD-SW difference is that the TD-SW during the last digits might be associated with a sustained encoding process for consolidating the digit groups rather than the preparatory process or simple retention process during the first digits. Previous studies have shown that a positive TD-SW could reflect increased processing for deliberate and sustained attention allocation (Gevins et al., 1996) or central executive operation for updating information (Kiss et al., 1998) in WM, which would be required for the elaborative encoding process in LTM.

#### Self-Paced Test vs. Fixed-Pace Test Conditions

Importantly, a very similar pattern of digit-position effects on EEG measures was observed both in the self-paced test and the fixed-pace test conditions. The result supports the notion that the aforementioned framework to explain the effect of digit-position on the EEG measures is applicable to both the self-paced and the fixed-presentation rate conditions. While the participant was allowed to regulate the study times (for encoding digits) in the self-paced test, the encoding period was static in the fixed-pace test. Despite these experimental differences, the participant essentially used the same mnemonic technique (i.e., grouping digits with semantic codes) during both the fixed-pace test and the self-paced test as demonstrated by the protocol analysis, and the pattern of EEG findings was strikingly similar across conditions. Thus, the similar pattern of significant digit-position effects suggests that processing mechanisms occurring during the both test conditions was qualitatively similar.

Differences with regard to the presentation-pace effect and the interaction between digit-position and presentation-pace provided interesting additional information. First, the overall increased time-frequency power of the theta and delta measures during the self-paced test supports that brain activities associated with the encoding process might be more prominent during the self-paced test, because the self-paced test would allow the participant to focus on each step of the encoding processes without time pressure. More specifically, increased power during the self-paced test was observed most strongly for theta, significantly for delta, and nominally (but not significantly) for the TD-SW. This trend of presentation-pace effect is also consistent with the idea that the early encoding processing is more attention-related in the early EEG measures, particularly, in theta, while the late TD-SW appears to be more independent of attention-related processing. It is also worthwhile to note that the interaction of digit-position and presentation-rate was only significant in theta, which suggests that the increased attentional modulation in theta accentuated differences observed between the first and last digits.

In sum, with regard to presentation-pace effect and the interaction, we found a significant contrast between the early and late EEG measures, which may suggest that the cognitive processes reflected in the late TD-SW are different from those of the early measures. Particularly, the late TD-SW might be a better candidate for a direct electrocortical index of elaborative encoding process underlying superior memory performance, which is relatively independent from the increased attentional processing.

### Implication for Studying Neural Indices of Superior Memory

Taken together, our results suggest that the behavioral differences in encoding processes between first and last digits are also related to differences in EEG correlates of encoding, namely, differences in theta, delta, and TD-SW. The major contribution of the current study is that those neural indices can be more directly attributable to encoding process underlying superior memory performance as well as LTWM. Most previous brain-imaging studies on superior memory or LTWM have relied on the assumption that changes in brain activities would be observable when individuals engage in a memory task requiring them to use memory skill(s). According to the assumption, they have simply measured brain activity during different types of memory task (e.g., Williamon and Egner, 2004; Raz et al., 2009), and/or compared the neural activity of individuals exhibiting an exceptional memory to those of a control group (e.g., Maguire et al., 2003; Yin et al., 2015). But, these studies have paid less attention to the issue of how to pinpoint encoding and retrieval processes underlying superior memory performance while the subjects were conducting memory tasks in a continuous stream of various cognitive processes (Maguire, 2012). There have been only few attempts to combine fMRI data with verbal reports data (e.g., Spiers and Maguire, 2006, 2007b). In addition, previous studies on the role of theta activity in encoding and retrieval (Nyhus and Curran, 2010) have simply relied on an episodic memory task that involves multiple cognitive operations, and they have rarely focused on how to isolate specific brain correlates of the various memory subprocesses (e.g., encoding).

It should be noted that the mnemonists in Pan et al. (2017) showed an increased P2 component and high-alpha oscillation on digits at even positions. Pan et al. (2017) suggested that the increased P2 was likely to reflect early-stage attention allocation for mental imagery processing at even positions. It is inconsistent with our suggestion that the greater theta and delta activities in the first digits (i.e., digits at odd-position in Pan et al., 2017) might be an index of increase top–down attentional control. One possible explanation is that the difference in mnemonic strategy between our participant and the mnemonists in Pan et al. (2017). Our digit-span tasks was adapted to present a constant state of challenge at the limits of participant’s current performance level. It would make the participant not only grouped the digits but also combined the digit groups as one story (i.e., super-group) for encoding longer digit sequences at the limits of current performance. This mnemonic strategy is very similar to generating a retrieval structure for encoding the longer digit sequences in Chase and Ericsson (1981, 1982) original studies. Particularly, as discussed above, encoding the last digits would consist of a more complex stream of ongoing cognitive processes (i.e., maintaining, updating, or consolidating previous digits and digit-groups) when the participant received the last digit. Thus, the attentional process might not be isolated from the other processes, which results in making it difficult to capture from the last-digits. In Pan et al. (2017), the digit memory task consisting of 12 digits might be not challenging enough for the mnemonists to use more complex encoding processes and thus the attentional. Thus, the attentional process in itself might be more acute to be captured in the digits at even-positions. The differences between the current study and those in Pan et al. (2017) may also be due to differences in the time-frequency methodology by the two studies. Pan et al. (2017) quantified non-phase-locked (induced) power, whereas the current study investigated phase-locked (evoked) power. Future studies can consider the differential effects of phase- and non-phase-locked power on encoding processes and exceptional memory performance in the same individuals.

The current study provides an opportunity to go beyond those earlier studies. We first reproduced superior memory performance on digit-span task under a controlled laboratory situation, which allowed us to identify direct behavioral indices of encoding process underlying superior memory performance. Then we successfully found ERP indices corresponding to the behavior indices. We believe that the current paper is the first laboratory study that illustrated how ERPs can successfully be combined with behavioral indices revealed by protocol analysis of one’s verbal reports on thought processes. This fine-grained approach would permit new insights into capturing and studying neural mechanism mediating superior memory. Given the participant’s baseline lack of superior basic memory capacity and the constant state of challenge during the digit-span tasks, we particularly believe that neural mechanisms found in the current study are associated with the participant’s effort to develop superior memory at the limits of current performance rather than exceptional intellectual ability or structural brain differences. The current findings thus also shed light on the study of brain activities occurring while individuals attempts to develop expertise at the limits of their performance (Ericsson, 2003).

### Limitations and Future Directions

The current study is not without important limitations. First of all, the current results are based on a single case study and thus may not be entirely generalizable. It is possible that anyone who uses the similar mnemonic strategy would show the similar pattern of brain activities. We are also not able to control possible expectation effects during the training sessions. To address the limitations, future studies should first attempt to replicate these findings in a larger sample of individuals with the same design of training used in the current study. We also need to consider a control memory practice training that (1) keeps presenting the length of digit-sequence which is not challenging to their performance or (2) asks participants to use a different type of mnemonic strategy (e.g., rote rehearsal without using mnemonic association). A control group who receives a different type of cognitive intervention would be helpful for controlling possible expectation effects on the digit-span tasks. The possible future studies, including exploring phase- and non-phase-locked power, would also allow us to clarify our explanation on the difference with Pan et al. (2017). However, it should be emphasized that our primary focus was to reveal the qualitative differences between the first and the last digits (assuming the independence of observation between them) rather than its generalizability. More importantly, the current study provided a rare opportunity to directly investigate brain activities associated with the basis of superior memory, addressing the methodological difficulty in isolating a specific neural activity while various cognitive processes simultaneously occur.

Second, it is unclear whether the reported pattern of behavioral and EEG findings are specific to digit-span processing, or would instead be found across other memory domains (e.g., spatial or visual memory tasks). Third, while it is relatively difficult to accurately measure single-trial ERP activity, trial-level information may provide important information regarding variations in superior memory performance. For example, ERP activity during encoding may possibly differ between digits from successful trials and those from unsuccessful trials. Fourth, the current study did not examine EEG activity during the recall/retrieval period, which may offer insight into the brain correlates of superior memory retrieval processes. Future work is needed to address these questions.

Given recent work detailing the importance of functional connectivity as a mechanism of information transfer among distant brain regions during memory and executive functioning (Sauseng and Klimesch, 2008; Clayton et al., 2015), it would also be of interest to evaluate interregional connectivity during encoding to possibly elucidate brain networks related to superior memory performance. In addition, future studies should assess higher-frequency band activity (e.g., beta, gamma) given their likely role in memory formation and retrieval processes (for a review, see Sauseng and Klimesch, 2008). Source localization techniques (e.g., distributed source models, dipole modeling, beamforming) may help to isolate the relative contributions of cortical (e.g., prefrontal cortex) and subcortical (e.g., hippocampus) brain regions to the observed EEG measures during memory.

## Ethics Statement

A written informed consent form was obtained from the participant both for the purposes of research participation as well as for the publication of this study data. The study protocol was reviewed and approved by the FSU Human Subjects Committee Institutional Review Board.

## Author Contributions

J-SY, JH, and EB developed the idea for the study. J-SY and JH prepared the materials, collected the data, and conducted the statistical analyses. J-SY, JH, WB, YG, and EB drafted the manuscript. J-SY and JH contributed equally to this article and should both be considered first authors.

## Conflict of Interest Statement

The authors declare that the research was conducted in the absence of any commercial or financial relationships that could be construed as a potential conflict of interest.
